# A Replication Study for Genome-Wide Gene Expression Levels in Two Layer Lines Elucidates Differentially Expressed Genes of Pathways Involved in Bone Remodeling and Immune Responsiveness

**DOI:** 10.1371/journal.pone.0098350

**Published:** 2014-06-12

**Authors:** Christin Habig, Robert Geffers, Ottmar Distl

**Affiliations:** 1 Institute for Animal Breeding and Genetics, University of Veterinary Medicine Hannover (Foundation), Hannover, Germany; 2 Department of Cell Biology, Helmholtz Centre for Infection Research, Braunschweig, Germany; Rutgers University, United States of America

## Abstract

The current replication study confirmed significant differences in gene expression profiles of the cerebrum among the two commercial layer lines Lohmann Selected Leghorn (LSL) and Lohmann Brown (LB). Microarray analyses were performed for 30 LSL and another 30 LB laying hens kept in the small group housing system Eurovent German. A total of 14,103 microarray probe sets using customized Affymetrix ChiGene-1_0-st Arrays with 20,399 probe sets were differentially expressed among the two layer lines LSL and LB (FDR adjusted P-value <0.05). An at least 2-fold change in expression levels could be observed for 388 of these probe sets. In LSL, 214 of the 388 probe sets were down- and 174 were up-regulated and vice versa for the LB layer line. Among the 174 up-regulated probe sets in LSL, we identified 51 significantly enriched Gene ontology (GO) terms of the biological process category. A total of 63 enriched GO-terms could be identified for the 214 down-regulated probe sets of the layer line LSL. We identified nine genes significantly differentially expressed between the two layer lines in both microarray experiments. These genes play a crucial role in protection of neuronal cells from oxidative stress, bone mineral density and immune response among the two layer lines LSL and LB. Thus, the different regulation of these genes may significantly contribute to phenotypic trait differences among these layer lines. In conclusion, these novel findings provide a basis for further research to improve animal welfare in laying hens and these layer lines may be of general interest as an animal model.

## Introduction

Whether and to what extent cellular development, differentiation, function and physiology occur does essentially depend on the repertoire, level and time point of expressed genes [1]. Therefore, experimental tools for direct and simultaneous monitoring of the expression levels of large numbers of genes in parallel are of great importance [1]. Affymetrix was the first company that developed a method to analyse large fragments and even entire genomes, called DNA microarrays [2]. Currently, the Affymetrix GeneChip microarrays are the most frequently used high-throughput technology to measure gene expression [3]. Microarrays are now available for a variety of different species, including agriculturally important species [4] like the chicken. Since the completion of the chicken genome sequence, assembly and annotation, as well as the availability of new high-throughput tools for the exploration of functional elements of the genome, the chicken became an important model organism [5], [6] for evolutionary and developmental biology, immunology, genetics and agricultural science [7].

Microarray technologies enable the examination of expression levels of thousands of genes in a single hybridization experiment. Microarray analyses can permit the understanding of biological pathways important in various physiological processes [4] and may therefore contribute to improvements in important economic parameters in poultry, like egg production and reproduction traits in ducks or feed efficiency and growth in broilers [8]–[10]. Furthermore, behavioural disorders, like feather pecking, a serious animal welfare and economically important problem, especially in the laying hen husbandry has been examined by using microarray analyses in several studies [11]–[13]. Labouriau et al. [11] used 20K chicken oligonucleotide microarrays (ARK-Genomics) to compare gene transcription profiles of a high feather pecking White Leghorn line, selected for eight generations, with another group of birds performing feather pecking. Among a total of 14,077 investigated genes they identified 456 genes to be differentially expressed between these two groups of laying hens, supporting their evidence of the presence of a major dominant allele affecting feather pecking behaviour. Differences in feather pecking behaviour between the two widespread commercial layer lines Lohmann Selected Leghorn (LSL) and Lohmann Brown (LB) are also confessed from several studies [12]–[13]. Due to these behavioural differences, as well as further differences in phenotypic traits, LSL and LB laying hens are not equally suitable for different housing systems. In a previous study, we identified 6,276 microarray probe sets to be significant differentially expressed (FDR adjusted P-value <0.05) between the two layer lines LSL and LB [14]. Among these probe sets, 151 had a 2-fold change or greater in gene expression and could be assigned to immune system processes and phosphorus metabolic processes. In order to confirm the conclusions drawn from this microarray experiment, a replication study was performed to validate the reliability of the results, as well as to improve the sensitivity and specificity of our previous analyses.

Therefore, the aim of the present study was to replicate the previous study under the very similar conditions and determine which genes are differentially expressed in both experiments.

## Results

### Phenotypic traits

For the phenotypic traits the least square means (LSM) with their standard errors (SE) and the P-values for differences between the two layer lines LSL and LB in the present experiment are given in Table 1. Although the egg weights were approximately equal in both layer lines, LSL hens laid eggs with significantly higher albumen heights and Haugh units, heavier yolks and eggshells, but at the same time thinner eggshells. At each time of examination, LSL layers were scored significantly worse for the plumage condition of the different body regions, resulting in a worse total plumage score compared to LB. The body weights, bone weights, as well as the bone lengths were significantly higher in LB than in LSL. Furthermore, LB hens had significantly higher tibia and humerus bone breaking strengths than LSL. The calculated heterophil to lymphocyte ratio (H/L-ratio) of LB layers was approximately 3-times higher than the average H/L-ratio determined for LSL.

**Table 1:** Least-square means (LSM), their standard errors (SE) and P-values for the phenotypic traits analysed in the two layer lines Lohmann Selected Leghorn (LSL) and Lohmann Brown (LB).

Trait	LB		LSL		LB - LSL
	LSM	SE	LSM	SE	P-value
Egg quality traits					
Egg weight (g)	63.99	0.23	64.40	0.23	0.210
Eggshell weight (g)	6.51	0.04	6.79	0.04	<0.001
Eggshell breaking strength (N)	41.29	0.46	40.00	0.46	0.058
Albumen height (mm)	8.45	0.07	9.23	0.07	<0.001
Haugh units	90.29	0.41	94.41	0.41	<0.001
Yolk weight (g)	16.07	0.07	16.99	0.07	<0.001
Eggshell thickness (µm)	343.14	1.12	338.48	1.11	0.007
Plumage condition (1–4)					
-Neck	2.74	0.05	2.45	0.05	<0.001
-Back	3.63	0.12	2.56	0.12	<0.001
-Wings	2.53	0.07	2.43	0.07	0.321
-Tail	2.48	0.05	1.85	0.05	<0.001
-Breast	2.32	0.04	1.62	0.04	<0.001
-Belly	3.38	0.07	1.99	0.07	<0.001
-Total	17.09	0.34	12.91	0.34	<0.001
Body weight (kg)	1.91	0.01	1.58	0.01	<0.001
Bone length (cm)					
-Tibia (cm)	11.85	0.03	11.58	0.02	<0.001
-Humerus (cm)	7.90	0.02	7.60	0.01	<0.001
Bone weight (g)					
-Tibia (g)	11.59	0.09	8.81	0.08	<0.001
-Humerus (g)	4.52	0.05	3.43	0.05	<0.001
Bone breaking strength (N)					
-Tibia (N)	146.78	3.15	137.29	3.13	0.044
-Humerus (N)	237.83	3.57	185.68	3.52	<0.001
H/L-ratio	0.84	0.05	0.29	0.05	<0.001

### Gene expression analysis

A total of 388 probe sets with at least a 2-fold change in expression could be assigned among the 14,103 probe sets. In LSL, 214 of the 388 probe sets were down- and 174 were up-regulated (Table S1). By contrast, the 214 down-regulated probe sets were enriched and the 174 highly expressed probe sets were down-regulated in the LB layer line (Figure S1). GO annotation of biological process was only given for 45 of the 388 significant probe sets. Among the 174 up-regulated probe sets in LSL, we identified 51 significantly enriched GO-terms of the biological process category (Table S2). Figure S2 shows a tree graph, representing the hierarchical relationships of these enriched GO-terms. A total of 63 enriched GO-terms could be identified for the 214 down-regulated probe sets of the layer line LSL. Relationships between these GO-terms are represented in Figure S3.

We compared the outcomes for the differentially expressed probe sets among the two layer lines of the present study with our previous microarray analysis for the same layer lines. A 2-fold or greater change in gene expression was identified on 151 probe sets in the previous experiment [14]. These 151 probe sets could be assigned to 79 different genes. The 388 significant probe sets of the present experiment could be assigned to 264 different genes. A comparison of all genes (n = 14,338) assigned to the probe sets of both microarray experiments revealed that 66% of the genes were in common. A total of 2343 genes of the previous microarray experiment were not represented in the present experiment, whereas 2605 genes of the present experiment were missing in the previous experiment. Among the differentially expressed genes from both experiments (n = 334), there were 301/334 genes (90%) on both microarray platforms represented. We found nine genes to be differentially expressed between the two layer lines in both experiments (Table 2). Assuming the same rate of differentially expressed genes (9/301 = 0.03) for the genes not present on both microarray platforms, we assume that we may have missed 0.03×65 = 1.95 genes potentially significantly differentially expressed. The probability for observing nine differentially expressed genes in the two experiments was P<0.0001.

**Table 2:** List of genes that were significant differentially expressed between the two layer lines Lohmann Selected Leghorn (LSL) and Lohmann Brown (LB) in both microarray experiments.

Gene	Gene	Chr	Chromosome	Chromosome	Regulation	Number of	Fold
symbol			start index	stop index	in LSL versus LB	transcripts	change
*AKR7A2*	*Aldo-keto reductase family 7,*	21	4669151	4673134	Up	2	2.24
	*member A2 (aflatoxin aldehyde reductase)*						2.17
*C20ORF177*	*chromosome 20 open reading frame*	20	7219219	7222018	Up	1	7.39
*COMTD1*	*Catechol-O-methyltransferase domain containing 1*	6	14390582	14396766	Down	1	-2.53
*IGJ*	*Immunoglobulin J polypeptide, linker protein for immunoglobulin*	4	49429222	49434990	Up	3	2.23
	*alpha and mu polypeptides*						2.42
							2.88
*PTPN5*	*Protein tyrosine phosphatise, non-receptor type 5 (striatum-enriched)*	5	12065964	12145215	Up	4	2.09
							3.15
					Down		−2.01
							−3.29
*RNF207*	*Ring finger protein 207*	21	634031	638889	Down	1	−2.53
*RPL21*	*Ribosomal protein L21*	1	175570306	175577122	Up	1	2.46
*SYT1*	*Synaptotagmin 1*	1	39261286	39381655	Down	1	−2.14
*UPK1B*	*Uroplakin 1B*	1	80215467	80223594	Up	2	2.55
							2.83

Given are the gene symbols and names, their chromosomal positions in base pairs and the chromosomes (Chr), up- or downregulation in LSL versus LB layers, the number of transcripts per gene with their fold changes.

## Discussion

The present study confirmed the layer lines as the most important factor influencing gene expression levels. While for the group size and tier of the housing system no significant differentially expressed genes could be found, we identified a great number of genes with different expression among the layer lines LSL and LB. Although the type of microarray chip was not identical in the two microarray experiments, we identified a total of nine genes to be significant differentially expressed in the same direction of regulation between the two layer lines in both experiments. Identification of these nine genes among the huge number of several ten thousands of probe sets indicates a crucial role of their gene products within processes that are different between the two layer lines.

The *aldo-keto reductase family 7*, *member A2* (*AKR7A2*) was one of these genes with higher expression in LSL in both experiments. AKR7A2 is one of the aldo-keto reductase enzymes that are necessary for detoxication and reduction of endogenous and exogenous aldehydes in mammals [15], [16]. AKR7A2 plays a significant role in protecting the brain from oxidative stress, because of its protective function of cells against the cytotoxicity of products arised from lipid peroxidation and correspondingly DNA damage, as well as its contribution to lowering reactive oxygen species [16]. Furthermore, this succinic semialdehyde reductase is catalyzing the biosynthesis of the neuromodulator γ-hydroxybutyrate [15].

The expression of *catechol-O-methyltransferase domain containing 1* (*COMTD1*) was down-regulated in LSL in the first as well as in the second experiment. Its gene product the catechol-O-methyltransferase 1 is responsible for the O-methylation of catechol estrogens, physiologically important catecholamines and many other catechols [17]. A common genetic polymorphism in the orthologous human *COMT* gene is a functional G>A polymorphism, resulting in an amino acid substitution of valine to methionine at codon 158 [18]–[20]. In comparison to the valine variant, the methionine variant has a 3- to 4-fold lower enzymatic activity [19]–[21] and is also associated with individual thermolability of the enzyme [20]–[22]. A lower bone mineral density has been found in men being homozygous for the low-activity *COMT* allele [19]. The *COMT* genotype can be used as an independent predictor of areal bone mineral density in the total body and in all femur locations, but not in the spine [19]. An increase in sympathetic activity due to a decreased degradation of catecholamines in individuals has been associated with the low-activity *COMT* genotype [19]. This increased activity of the sympathetic nervous system has a catabolic effect on bone formation [23]. Therefore, these findings may explain the increased risk for osteoporotic fractures and for fragility fractures in human male carriers of the Met^158^ low-activity allele [18]. The low expression of the *COMTD1* gene in LSL in the current study may have resulted in low expression levels of the catechol-O-methyltransferase, and correspondingly a decrease in substrate conversion. Consequently, the LSL layers had a higher sympathetic activity, which might explain the significantly lower humerus and tibia bone breaking strengths measured in LSL compared to LB. Furthermore, the increase in sympathetic activity might be a reason for the significant differences of plumage condition between LSL and LB laying hens. The major reason for the observed damage of plumage up to bare regions, especially in LSL, could be found in feather pecking. This term describes a behaviour that involves hens destroying the plumage of their flock mates, in some cases even pulling out feathers and eating them [24]. Significantly higher plasma noradrenaline levels and a faster heart rate during manual restraint, both indicative for a high sympathetic reactivity, in high feather peckers have been reported for a White Leghorn line compared to low feather peckers of the same layer line [25], [26].

The up-regulation of *EXT2* in LSL may also be contributed to their lower bone breaking strengths. *EXT2* encodes for exostosin 2, an enzyme that synthesizes heparan sulfate proteoglycans [27]. In human, a genetic mutation in exostosin 2 is associated with skeletal syndromes or predispositions to certain skeletal diseases [28].

Another gene with different expression between the two layer lines in both studies was *IGJ*, encoding the immunoglobulin J polypeptide, linker protein for immunoglobulin alpha and mu polypeptides. Beside the monomer IGM, IGJ is one of the components that mediate the B-cell-specific expression of IGM antibodies [29], and therefore plays an important role in the immune response. In LB, lower expression could be found for one transcript variant of IGJ in the first and even three transcript variants in the second experiment. In agreement with previous results, we attribute this down-regulation of immune system components to the elevated stress levels seen in LB [14]. Layers of the LB line showed almost 3-fold higher heterophil to lymphocyte (H/L) ratios than LSL hens. The measurement of the H/L-ratio is a common method to detect mild to moderate avian stress [30], which causes a heterophilia and a corresponding raised H/L-ratio [31]. Following a scale suggested by Gross and Siegel [32], LB layers of the present study have been exposed to long-term and high levels of stress, while LSL hens showed mean H/L-ratios of 0.29, indicative for low levels of stress. It is well known that the influence of stress causes a down-regulation of immune responsiveness [33]–[38]. The high stress levels measured for LB may therefore have suppressed the gene expression of *IGJ*.

In conclusion, the results of the two microarray studies in layer lines have shown *COMTD1*, *AKR7A2* and *IGJ* as strong candidates causing phenotypic differences in bone breaking strength, stress levels and immune responsiveness among layer lines. These findings have large implications for improving animal welfare and demonstrate the chicken as a useful animal model for explaining these metabolic processes.

## Material and Methods

### Ethics statement

All animal work has been conducted according to the national and international guidelines for animal welfare. The Lower Saxony state veterinary office, Niedersächsisches Landesamt für Verbraucherschutz und Lebensmittelsicherheit, Oldenburg, Germany, was the responsible Institutional Animal Care and Use Committee (IACUC) for this specific study. Exsanguination of laying hens at the University of Veterinary Medicine Hannover was under the supervision of the Lower Saxony state veterinary office, Niedersächsisches Landesamt für Verbraucherschutz und Lebensmittelsicherheit, Oldenburg, Germany. The experiments for the present study have been approved by the Lower Saxony state veterinary office, Niedersächsisches Landesamt für Verbraucherschutz und Lebensmittelsicherheit, Oldenburg, as a notifiable experiment with the registration number 33.9-42502-05-11A154.

### Layer lines and housing system

The investigated flock of laying hens was kept in the small group housing system Eurovent German provided by Big Dutchman (Vechta, Germany). The housing system was installed at the farm for education and research of the University of Veterinary Medicine Hannover (Foundation) in Ruthe. Investigations have been carried out on the duration of one laying period, starting in October 27, 2010 and ending in October, 14, 2011. The housing system comprised three tiers with ten compartments each. The two commercial layer lines, Lohmann Selected Leghorn (LSL) and Lohmann Brown (LB) were kept in group sizes of 36 and 54 hens per compartment. Each tier consisted of five successive compartments of LSL and another five of LB, which were arranged in alternate order. The layer lines were equally distributed among the two group sizes, but not mixed within a single compartment. All compartments had an equal height and depth of 60 cm and 135 cm, respectively. According to the different group sizes, the compartments were 240 and 362 cm wide, respectively. Hence, the available floor space per hen was 890 cm^2^. The total flock size was 1350 laying hens, with 684 LB and 666 LSL hens. After a rearing period of 18 weeks in cages the birds were moved to the small group housing system. The equipment of each compartment comprised dust baths, nest boxes with flexible curtains, claw abrasion devices, perches and a manure belt ventilator. Per compartment, two white plastic and another two metal perches, as well as the central tube for the automatic distribution of dust bathing substrate cold be used for perching by the hens. The four perches were installed in a stepped position at two different heights of 9 cm and 28 cm. The perch length per hen was 15 cm. The distribution of the layer lines and group sizes within the housing system and the structure of a single compartment in cross section and top view are shown in Figure S4. The lighting period was gradually stepped up until reaching 14 hours of light at the fifth week of the laying period. Both layer lines had an approximately equal egg production per hen housed (78%), with an average egg mass per hen housed of around 19 kg. Even the egg production (83%) and egg mass (21 kg) per average hen were similar in LSL and LB. With 3.91%, dirty eggs were more common in LSL hens than in LB (2.56%). In LSL, as well as in LB almost 4% of the eggs were cracked. While the overall mortality was approximately 4% for LB, almost 6% of the LSL laying hens died or had to be culled during the laying period.

### Management and feeding

All hens had identical feeding and management conditions throughout the whole laying period. A three-phase feeding program was provided to cover the amounts needed of calcium, phosphor and energy. Feed and water were provided ad libitum. A standard vaccination program for laying hens was applied during the rearing and laying period.

### Phenotypic traits

A total of 480 eggs each were randomly sampled in the 3^rd^, 9^th^ and 12^th^ laying month and recorded for the following internal and external egg quality traits including egg weight, eggshell breaking strength, albumen height, Haugh units, yolk weight, eggshell thickness and eggshell weight.

In the 2^nd^, 8^th^ and 13^th^ laying month the plumage condition of 480 laying hens each was scored using an evaluation scheme comprising four scores. A massive damage of the plumage and bare regions was assessed with Score 1. Score 2 was given when an explicit damage of feathers and/or bare areas could be observed. Completely or nearly complete feathered birds, with only a few damaged feathers were scored with 3 and score 4 was given for a very good plumage condition with nearly no damaged feathers. The six body regions neck, breast, belly, back, wings and tail were scored separately and added to a total plumage score. Furthermore, all hens were weighed using a digital table scale.

Approximately 0.5 ml of blood was drawn from a wing vein of 360 hens out of the 480 birds investigated in the 13^th^ laying month. Blood samples were used for preparing two methanol-fixed and one native blood smear within 30 minutes after sampling. After staining according to a modified Wright-Giemsa protocol, at least 400 leucocytes, including heterophils, lymphocytes, monocytes, basophils and eosinophils were counted on one slide per hen. The H/L-ratios were calculated by dividing the relative numbers of heterophils by the relative numbers of lymphocytes.

The 360 laying hens were subsequently transported to the Clinic for Poultry of the University of Veterinary Medicine Hannover (Foundation), where they were sacrificed by exsanguination after stunning them by rabbit punch. For further analyses of bone breaking strengths alternately the intact right or left *humeri* and *tibiae* were dissected and removed from the tissue surrounding the bones. Initially all bones were frozen at −20°C and examined after thawing within the next four weeks. After determination of the bone lengths and weights (in cm and g, respectively), the bone breaking strengths were measured in Newton (N) using a three-point-bending machine (Zwick/Z2.5/TNIS, Zwick-Roell, Ulm, Germany), which was controlled and calibrated by the technical service of Zwick-Roell in regular intervals. The punching tool exerts a constant, perpendicular force on the midshaft of the bone until its fracture.

### Statistical analysis of the phenotypic traits

Statistical analyses were performed using SAS, version 9.3 (Statistical Analysis System Institute Inc., Cary, North Carolina, USA). Egg quality traits, plumage condition, blood parameters and bone traits were analysed employing the procedure MIXED. Analysis of variance was applied after normal distribution of the logarithmically transformed white blood cell numbers and H/L-ratios, as well as the residuals of bone traits were confirmed using the Shapiro-Wilk and Kolmogorov-Smirnov tests of the UNIVARIATE procedure of SAS. The group size, tier, layer line and the interactions between layer line and group size, layer line and tier were regarded as fixed effects in the statistical model for the blood parameters and bone traits. The statistical model for the egg quality traits and the plumage condition additionally comprised the laying month as a fixed effect, as well as its interaction with the layer line. The individual compartments within layer line and trial were treated as randomly distributed effects. F-tests were conducted to examine whether an effect in the statistical model was significant or not, with a level of significance of P<0.05. A normal distribution was given for the residuals of all traits examined.

Statistical model for the blood parameters and bone traits:



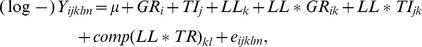
where Y_ijklm_ = white blood cell numbers, H/L-ratios and bone traits, μ = model constant, GR_i_ = fixed effect of group size (i = 1 to 2), TI_j_ = fixed effect of tier (j = 1 to 3), LL_k_ = fixed effect of layer line (k = 1 to 2), LL*GR_ik_ = fixed effect of interaction between layer line and group size, LL*TI_jk_ = fixed effect of interaction between layer line and tier, comp(LL*TR)_kl_ = randomly distributed effect of compartment within layer line (LL) and trial (TR), e_ijklm_ = random error variation.

Statistical model for the egg quality traits and the plumage condition:



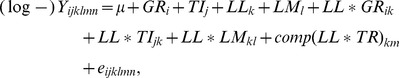
where LM_l_ = fixed effect of laying month, LL*LM_kl_ = fixed effect of interaction between layer line and laying month.

### Sampling and RNA isolation

When choosing hens for microarray analysis we selected between stressed and unstressed birds based on their behaviour during handling, their plumage condition and the number of skin lesions. In consideration of the group sizes, the tiers and the layer lines two to three hens per compartment were chosen for sampling, giving a total of 70 layers. After the hens were stunned and sacrificed by exsanguination, samples of four different regions of the cerebrum were taken from each hen at necropsy; one from the anterior left, one from the anterior right and another two from the posterior left and right side, respectively. All samples were extracted without meninges and immediately stored in 1.5 ml RNAlater stabilization solution (Qiagen, Hilden, Germany). As substitute sample of the remained cerebrum was stuck into cryogenic tubes and snap frozen in liquid nitrogen. The time from slaughtering to storing the samples was less then 15 minutes. RNAlater-samples were kept at +4°C for 24 hours and afterwards stored with the supplementary samples at −80°C. After thawing, approximately 50 mg tissue per sample were removed from RNAlater and transferred into microcentrifuge tubes (2 ml) containing 1 ml of QIAzol Lysis Reagent (Qiagen). Samples were disrupted and homogenized using 5 mm stainless steel beads (Qiagen) on a Tissue Lyser (Qiagen). The extraction of total RNA from the tissues was performed using the RNeasy Lipid Tissue Mini Kit (Qiagen) as described by the manufacturer, including the additional step of DNA-digestion. The total RNA concentration was measured using a NanoDrop 1000 spectralphotometer (Peqlab Biotechnologie, Erlangen, Germany).

### Synthesis of cDNA and microarray hybridization

Microarray analyses of 60 samples from the posterior left and right site of the cerebrum were performed by Helmholtz Centre for Infection Research (HZI), Braunschweig, Germany. In a first step quality and integrity of total RNA was assessed using the Agilent Technologies 2100 Bioanalyzer (Agilent Technologies, Waldbronn, Germany). Target preparation for RNA microarray processing was carried out according to the Ambion WT Expression Kit. A total of 500 ng RNA were used for biotin labelling according the Ambion WT Expression Kit (Ambion, Austin, TX). 5.5 µg of fragmented biotinylated cRNA were placed in a hybridization cocktail containing four biotinylated hybridization controls (BioB, BioC, BioD and Cre) as recommended by the manufacturer. Samples were hybridized to an identical lot of Affymetrix ChiGene-1_0-st Arrays (Affymetrix) for 17 hours at +45°C. After hybridization the microarrays were washed and stained with Streptavidin Phycoerythrin on the Fluidics Station 450 using the FS450-0002 protocol (Affymetrix). Finally, microarrays were scanned by the Affymetrix GCS3000 Scanner (Affymetrix) and the scanned images were analyzed with GCOS1.2 Software Suite (Affymetrix).

### Analysis of microarray data

Each Affymetrix microarray contained 20,399 probe sets. The GeneSpring GX 11 Software (Agilent Technologies, Santa Clara, California, USA) was used for statistatical analysis. Raw microarray data were normalized using the Robust Multi-array Average method (RMA). After background correction data were transformed and quantile global normalized at probe level to the median using a non-linear algorithm. To sort out all the unexpressed genes, raw data were filtered according to expression levels by cut off the 20^th^ percentile. Analyses for the effects layer line, group size and tier were performed using an unpaired T-test with multiple testing of Benjamini and Hochberg. The accepted level of significance was P<0.05. Genes with a 2-fold change or greater in gene expression were considered differentially expressed. We used the Gene Ontology (GO) Enrichment Analysis Toolkit (GOEAST, http://omicslab.genetics.ac.cn/GOEAST) to identify significantly enriched GO-terms among the lists of differentially expressed genes. The hypergeometric test method with Benjamini and Hochberg as multi-test adjustment method and FDR<0.1 as significance level of enrichment were selected for analyses. A GO-term was considered significantly enriched, if it could be assigned to at least two or more genes. The GO comprises three categories, named biological process, molecular function and cellular component. Within each of these categories the specific terms are considered children of more broad terms (child-parent relationships) [39]. For the current study the analyses only were made for terms belonging to the biological process of gene ontology. All significantly expressed probe sets were cross-checked against the data of the National Center for Biotechnology Information (National Center for Biotechnology Information (NCBI), http://www.ncbi.nlm.nih.gov/) and the chicken genome assembly (AmiGo, http://amigo.geneontology.org/cgi-bin/amigo/go.cgi).

### NCBI GEO submission

The normalized data from the present microarray gene expression experiment has been submitted to NCBI's Gene Expression Omnibus (GEO, http://www.ncbi.nlm.nih.gov/geo/) and can be queried via GEO series accession number GSE55570 (http://www.ncbi.nlm.nih.gov/geo/query/acc.cgi?acc=GSE55570). The previous data set can be read via the GEO accession number GSE40802.

## Supporting Information

Figure S1 **Heat map of differentially expressed probe sets among the two layer lines.** Heat map of the probe sets with absolute fold changes of 2-fold or greater detected in the comparison between the layer lines Lohmann Brown (LB) and Lohmann Selected Leghorn (LSL). The range of relative expression levels from lowest to highest is represented by the blue and red dyeing, respectively.
(JPG)

Figure S2 **Tree graph of the enriched Gene ontology (GO) terms of the biological process category among the list of probe sets with higher expression in LSL (GOEAST,** http://omicslab.genetics.ac.cn/GOEAST). The tree graph displays the hirachial relationships of biological processes identified to be enriched among the list of probe sets with given gene symbol that were significantly up-regulated in the layer line LSL. Significantly enriched GO terms are marked yellow. GO terms without significance are either shown as white boxes or drawn as points. Relationship between two enriched GO terms are marked with red edges, black solid edges stand for relationship between enriched and unenriched terms, black dashed edges stand for relationship between two unenriched GO terms. Each box represents a GO term, labeled by its GOID, the term definition, the P-value and detail informations.
(PDF)

Figure S3 **Tree graph of the enriched Gene ontology (GO) terms of the biological process category among the list of down-regulated probe sets in LSL (GOEAST,** http://omicslab.genetics.ac.cn/GOEAST). The tree graph displays the hirachial relationships of biological processes identified to be enriched among the list of probe sets with given gene symbol that were significantly up-regulated in the layer line LSL. Significantly enriched GO terms are marked yellow. GO terms without significance are either shown as white boxes or drawn as points. Relationship between two enriched GO terms are marked with red edges, black solid edges stand for relationship between enriched and unenriched terms, black dashed edges stand for relationship between two unenriched GO terms. Each box represents a GO term, labeled by its GO-ID, the term definition, the P-value and detail informations.
(PDF)

Figure S4 **Arrangement and dimensions of the compartments of the small group housing system.** **A** Cross section drawing of a single compartment. **B** Individual compartment for group sizes of 54 laying hens in a top view drawing. **C** Arrangement drawing of the tiers (A: first tier; B: second tier; C: third tier), layer lines (LB: Lohmann Brown; LSL: Lohmann Selected Leghorn) and group sizes (36 and 54 hens) of the small group housing system Eurovent German.
(TIF)

Table S1 **Differentially expressed probe sets between the Lohmann Selected Leghorn (LSL) and Lohmann Brown (LB) laying hens.**
(XLS)

Table S2 Enriched biological processes of probe sets with different expression in comparison between LSL and LB.
(XLS)
