# Supplementary figures and images for: A Replication Study for Genome-Wide Gene Expression Levels in Two Layer Lines Elucidates Differentially Expressed Genes of Pathways Involved in Bone Remodeling and Immune Responsiveness
Source: PLoS One. 2014 Jun 12;9(6):e98350. doi: 10.1371/journal.pone.0098350 (PMC4055560; doi:10.1371/journal.pone.0098350)

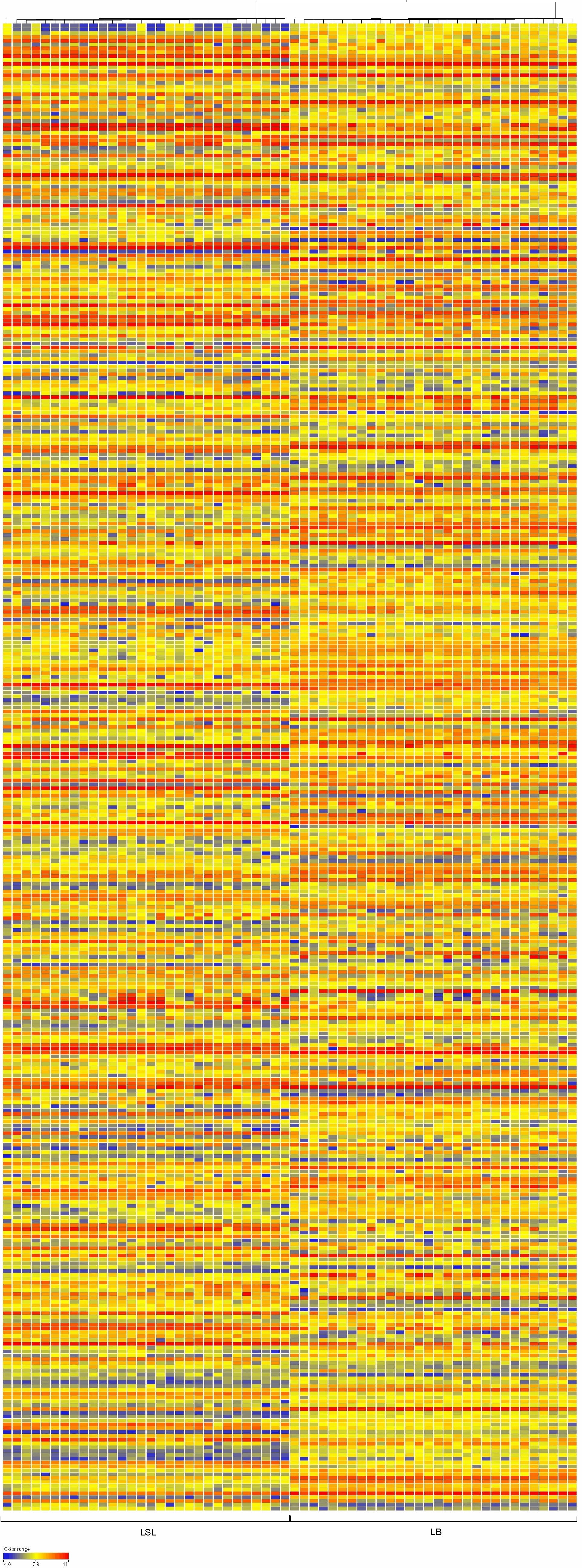

Supplement: Figure S1 — Heat map of differentially expressed probe sets among the two layer lines. Heat map of the probe sets with absolute fold changes of 2-fold or greater detected in the comparison between the layer lines Lohmann Brown (LB) and Lohmann Selected Leghorn (LSL). The range of relative expression levels from lowest to highest is represented by the blue and red dyeing, respectively. (JPG) [file pone.0098350.s001.jpg]

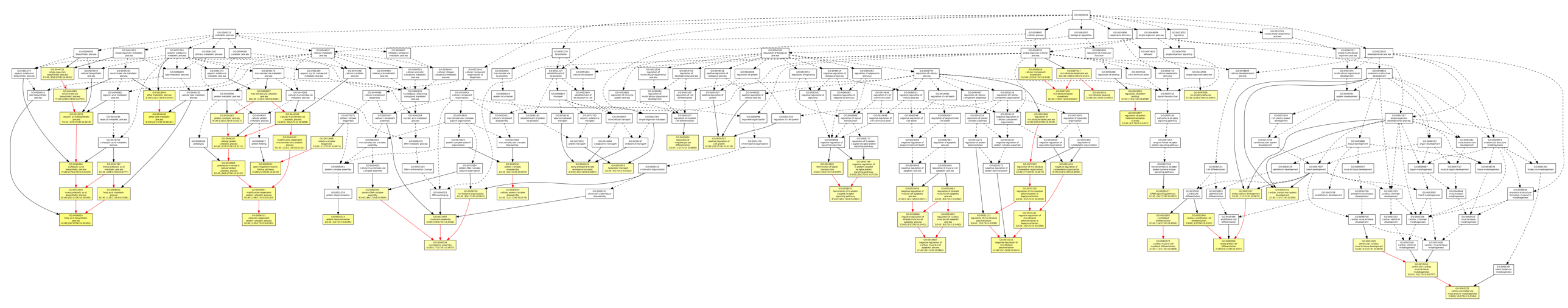

Supplement: Figure S3 — Tree graph of the enriched Gene ontology (GO) terms of the biological process category among the list of down-regulated probe sets in LSL (GOEAST, http://omicslab.genetics.ac.cn/GOEAST). The tree graph displays the hirachial relationships of biological processes identified to be enriched among the list of probe sets with given gene symbol that were significantly up-regulated in the layer line LSL. Significantly enriched GO terms are marked yellow. GO terms without significance are either shown as white boxes or drawn as points. Relationship between two enriched GO terms are marked with red edges, black solid edges stand for relationship between enriched and unenriched terms, black dashed edges stand for relationship between two unenriched GO terms. Each box represents a GO term, labeled by its GO-ID, the term definition, the P-value and detail informations. (PDF) [file pone.0098350.s003.pdf]

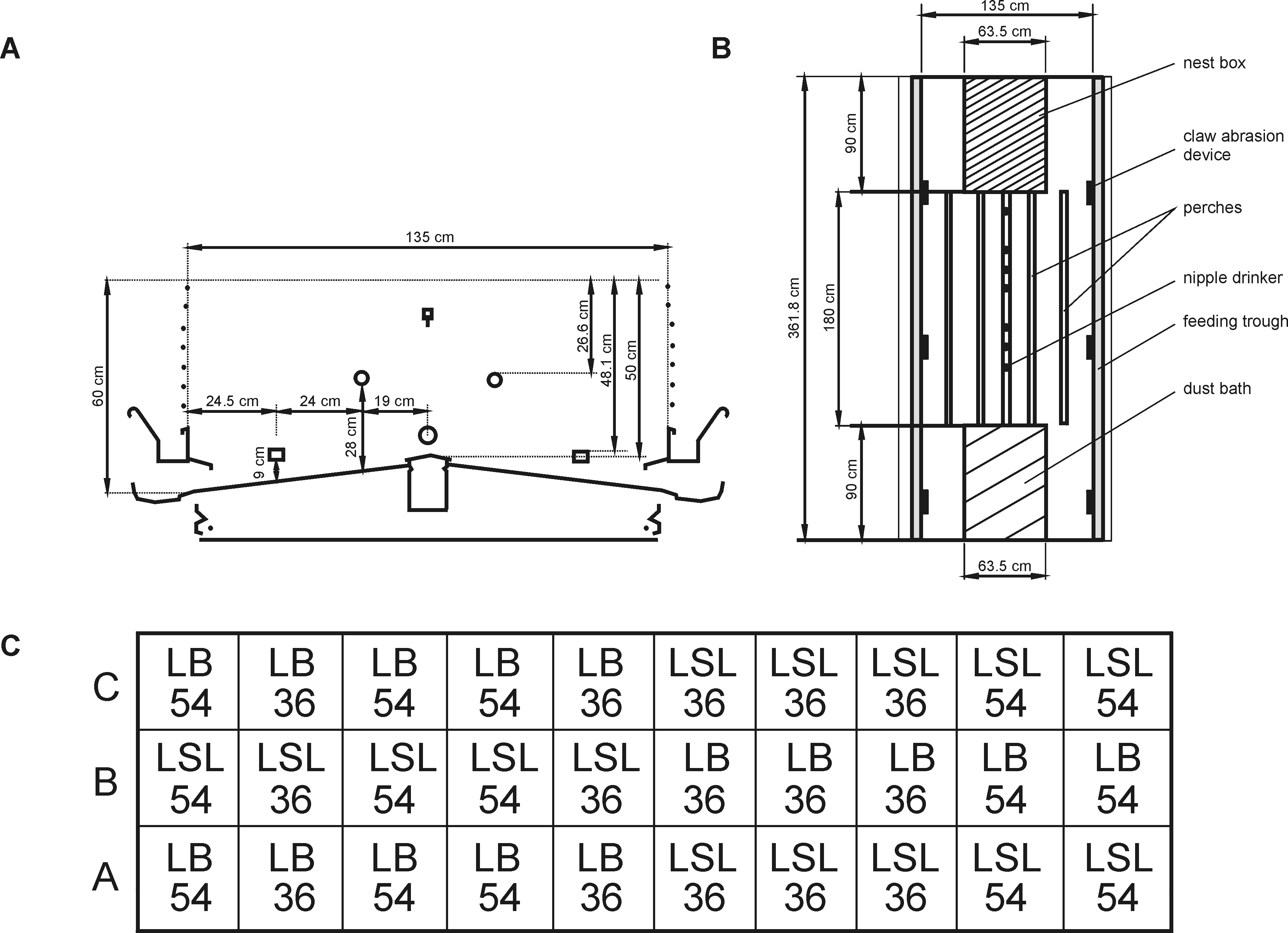

Supplement: Figure S4 — Arrangement and dimensions of the compartments of the small group housing system. A Cross section drawing of a single compartment. B Individual compartment for group sizes of 54 laying hens in a top view drawing. C Arrangement drawing of the tiers (A: first tier; B: second tier; C: third tier), layer lines (LB: Lohmann Brown; LSL: Lohmann Selected Leghorn) and group sizes (36 and 54 hens) of the small group housing system Eurovent German. (TIF) [file pone.0098350.s004.tif]
