# Supplementary material for: A Replication Study for Genome-Wide Gene Expression Levels in Two Layer Lines Elucidates Differentially Expressed Genes of Pathways Involved in Bone Remodeling and Immune Responsiveness
Source: PLoS One. 2014 Jun 12;9(6):e98350. doi: 10.1371/journal.pone.0098350 (PMC4055560; doi:10.1371/journal.pone.0098350)

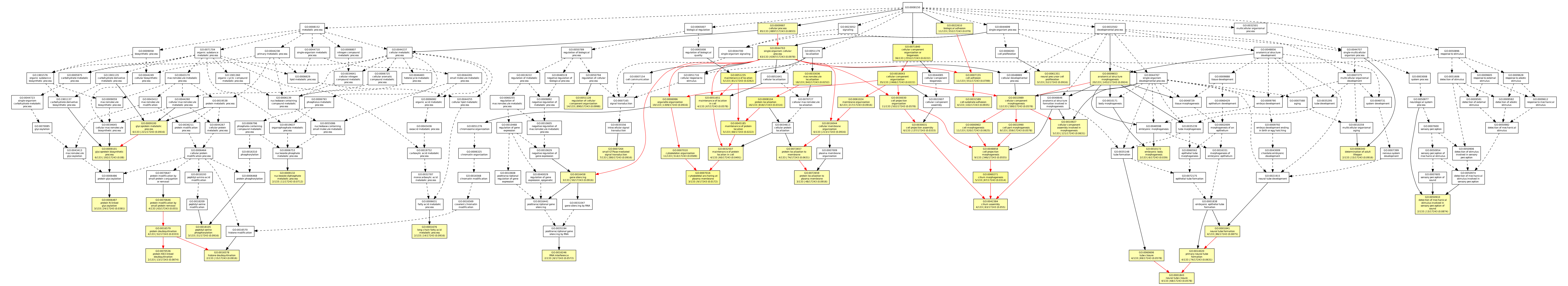

Supplement: Figure S2 — Tree graph of the enriched Gene ontology (GO) terms of the biological process category among the list of probe sets with higher expression in LSL (GOEAST, http://omicslab.genetics.ac.cn/GOEAST). The tree graph displays the hirachial relationships of biological processes identified to be enriched among the list of probe sets with given gene symbol that were significantly up-regulated in the layer line LSL. Significantly enriched GO terms are marked yellow. GO terms without significance are either shown as white boxes or drawn as points. Relationship between two enriched GO terms are marked with red edges, black solid edges stand for relationship between enriched and unenriched terms, black dashed edges stand for relationship between two unenriched GO terms. Each box represents a GO term, labeled by its GOID, the term definition, the P-value and detail informations. (PDF) [file pone.0098350.s002.pdf]
